# Identification of QTLs conferring resistance to scald (*Rhynchosporium commune*) in the barley nested association mapping population HEB-25

**DOI:** 10.1186/s12864-020-07258-7

**Published:** 2020-11-27

**Authors:** Bianca Büttner, Vera Draba, Klaus Pillen, Günther Schweizer, Andreas Maurer

**Affiliations:** 1 Bavarian State Research Center for Agriculture, Institute for Crop Science and Plant Breeding, Freising, Germany; 2 Martin Luther University Halle-Wittenberg, Institute of Agricultural and Nutritional Sciences, Chair of Plant Breeding, Halle, Germany

**Keywords:** HEB-25, *Hordeum vulgare*, *Hordeum vulgare ssp. spontaneum*, *Rrs*, Wild barley, Scald resistance, *Rhynchosporium commune*, Nested association mapping (NAM), Greenhouse trials

## Abstract

**Background:**

Barley scald, caused by the fungus *Rhynchosporium commune*, is distributed worldwide to all barley growing areas especially in cool and humid climates. Scald is an economically important leaf disease resulting in yield losses of up to 40%. To breed resistant cultivars the identification of quantitative trait loci (QTLs) conferring resistance to scald is necessary. Introgressing promising resistance alleles of wild barley is a way to broaden the genetic basis of scald resistance in cultivated barley. Here, we apply nested association mapping (NAM) to map resistance QTLs in the barley NAM population HEB-25, comprising 1420 lines in BC_1_S_3_ generation, derived from crosses of 25 wild barley accessions with cv. Barke.

**Results:**

In scald infection trials in the greenhouse variability of resistance across and within HEB-25 families was found. NAM based on 33,005 informative SNPs resulted in the identification of eight reliable QTLs for resistance against scald with most wild alleles increasing resistance as compared to cv. Barke. Three of them are located in the region of known resistance genes and two in the regions of QTLs, respectively. The most promising wild allele was found at *Rrs17* in one specific wild donor. Also, novel QTLs with beneficial wild allele effects on scald resistance were detected.

**Conclusions:**

To sum up, wild barley represents a rich resource for scald resistance. As the QTLs were linked to the physical map the identified candidate genes will facilitate cloning of the scald resistance genes. The closely linked flanking molecular markers can be used for marker-assisted selection of the respective resistance genes to integrate them in elite cultivars.

**Supplementary Information:**

The online version contains supplementary material available at 10.1186/s12864-020-07258-7.

## Background

*Rhynchosporium commune*, a haploid fungus, is the causal agent of scald or barley leaf blotch, an important foliar disease of barley (*Hordeum vulgare ssp. vulgare* L.). *R. commune* has been classified as a hemibiotroph fungus which occurs in all barley growing areas around the world, especially in cool, humid climates [1]. The typical disease symptoms are tan necrotic lesions with dark brown margins, which occur after a latent period [2]. Besides barley the genus *Rhynchosporium* is able to infect further species, e. g. rye [1] and *Lolium* species [3]. Scald can cause yield losses of up to 40%, decreases grain quality [2, 4] and is considered a major economic barley disease, especially in the UK, Australia and Tunisia [5–7].

In practice the pathogen is mainly controlled by growing resistant cultivars or chemical protection, while phytosanitary techniques, e. g. ploughing or crop rotation can also help to protect the crop [1, 2]. In general, winter barley cultivars show a higher partial resistance to scald than spring barley cultivars [2]. Because the pathogen itself is highly diverse [1, 2, 8] scald is able to overcome crop protection methods like fungicides or specific cultivation methods as well as resistance genes within a few growing seasons, especially when extensively used [2]. In addition, the high genetic variation may enable the pathogen to cope with climate warming [9].

Since decades breeders and scientists deal with the complex interaction of barley and *R. commune*, which is only partly understood. Up to now, nine major resistance genes (R genes) and many quantitative trait loci (QTLs) have been identified and have been mapped on a consensus bin-map [2]. Björnstad et al. [10] suggested a nomenclature to classify R genes against *Rhynchosporium* using the *Rrs*/*rrs* terminology considering that some described resistance genes are alleles of the same gene. Part of the R gene suite identified thus far are derived from wild *Hordeum* species, e. g. *Rrs13*, *Rrs14* and *Rrs15* from *Hordeum vulgare ssp. spontaneum* [11–16] or *Rrs16* from *Hordeum bulbosum* [17]. Most of them are mapped and markers for marker-assisted selection (MAS) are developed, but except *Rrs2* [18] none of them are diagnostic. So far, none of the scald resistance genes has been cloned [1, 19].

The *Rrs1* locus was the first resistance locus described in barley, which is a powerful and still effective resistance locus against scald in barley [20], although the fungus is able to overcome *Rrs1* by losing the avirulence gene *NIP1* (necrosis-inducing peptide 1, [21]). *Rrs1* represents a complex locus with either many tightly linked genes or multiple alleles at a single gene allocated to the centromeric region of chromosome 3HL [2, 22]. Although *Rrs1* is a major resistance gene sometimes even in resistant plants the fungus is able to complete its life cycle and sporulate [23–25]. Additionally, Patil et al. [26] mapped a second resistance locus, named *Rrs4*_*CI11549*_ 22 cM distal to *Rrs1* on chromosome 3HL. For *Rrs2*, located on 7HS [27], eight diagnostic markers are described [18]. The genomic region near *Rrs2* was re-sequenced in a diverse set of wild and cultivated barley. The nucleotide diversity was higher in wild than cultivated barley and the domestication signal in this region was weak [28]. Pectin esterase inhibitor (PEI) genes were analysed as possible candidate genes for *Rrs2*, but this could not be confirmed [29]. *Rrs13* is derived from a wild barley accession and located on the short arm of chromosome 6H [12, 30]. In this region are several QTL for scald resistance [31–35]. *Rrs14* originated from a wild population of *Hordeum vulgare ssp. spontaneum* from Iran and is located between the seed storage protein loci *Hor1* and *Hor2* (hordein) on 1H [14]. Two different loci conferring resistance were both named *Rrs15* whereby one is located on 2H and the other on 7H [2]. The single dominant gene on the long arm of chromosome 7H is derived from an Israeli accession of wild barley [16]. The locus on 2H originated from *Hordeum vulgare* and is named *Rrs15*_*CI8288*_ according to the resistance donor CIho8288 [36]. To avoid confusion Zhan et al. [2] suggested to rename the locus on 2H as *Rrs17*. The first resistance gene against scald from the secondary gene-pool of barley is *Rrs16*^*Hb*^ on chromosome 4HS. It is derived from a recombinant hybrid between *H. vulgare* and *H. bulbosum* [17]. A recently identified resistance locus on chromosome 6H is called *Rrs18* and was mapped distal from *Rrs13* on chromosome 6H [37, 38].

Possibly some QTLs are alleles of known resistance genes [39]. Furthermore, some QTLs independently identified in different studies may be allelic or even identical [2]. Schweizer and Stein [40] integrated 166 QTLs from 28 studies to identify meta-QTLs mediating resistance to several fungal pathogens. Twenty meta-QTLs were detected over all chromosomes including ten regions associated with scald resistance on all chromosomes except chromosome 5H. Looseley et al. [19] identified altogether 17 QTLs by means of genome-wide association study (GWAS) in two different data sets (European spring barley and old list trials). The QTLs are in the region of *Rrs1*, *Rrs3*, *Rrs13*, *Rrs15b* and *Rrs16*, but are probably not the resistance genes. Wang et al. [41] combined in total 43 QTLs and seven genes conferring quantitative and qualitative resistance, which had been located on individual maps, in a consensus map. Again they found QTLs/genes on all chromosomes except 5H.

The majority of genetic studies on scald resistance has been conducted at the seedling stage focusing on major gene resistance presumably based on problems with the field tests [2, 42]. Normally there is a good correlation between seedling and adult plant resistance with some exceptions [2, 33]. Disease escape may be one reason why plants seem to be more resistant in the field than in the greenhouse [2]. Disease escape is mainly based on unfavourable growing conditions for the fungi like drought and temperature as well as plant height, maturity and canopy structure limiting the spreading of the pathogen [2]. Therefore, the resistance QTLs on 3H [32, 33, 43, 44] in the region of *sdw1* possibly are pleiotropic effects of the semi-dwarfing gene [2]. Adult plant resistance (APR) is based on many minor genes with small effects [41, 45]. Accordingly, several QTLs for APR were detected on chromosome 2H, 3H, 4H, 6H and 7H [31–33, 41–43, 46, 47].

During the last decade the concept of nested association mapping (NAM) was established as a method to identify QTLs with high precision and high statistical power by combining advantages of classical linkage mapping and association mapping. In NAM a multitude of highly divergent (exotic) parents are crossed with one recurrent elite cultivar. This way potentially useful exotic alleles can be investigated in an adapted background, which is of special importance for allele mining of exotic resistance genes. In this regard NAM has been successfully applied to identify exotic sources of pathogen resistance in maize [48–50], barley [51–53] and wheat [54, 55]. Vatter et al. [52, 53] could show that the wild barley NAM population HEB-25 contains numerous novel QTLs for net blotch, leaf rust and stripe rust. However, resistances were mostly conferred by the combination of multiple small-effect QTLs rather than single major QTLs.

The aims of the study were I) to screen the HEB-25 population for scald resistance; II) to detect QTLs linked to resistance against *R. commune*; III) to compare the identified QTLs with known resistance genes and QTLs; IV) and to find highly resistant HEB-25 lines for introgression of the resistance improving alleles in pre-breeding programs.

## Results

### Scald resistance

Of the 26 HEB parents only HID_138 (donor of HEB family 13 (F13)), HID_380 (F24) and Barke (recurrent parent) were clearly susceptible in the greenhouse tests. The accession HID_144 (F15) segregated for resistance and the remaining accessions were resistant. Fifteen accessions showed no symptoms at all and the other seven resistant accessions showed only small lesions (Additional file 1: Figure S1). All 25 HEB families were segregating for scald resistance. HEB family 01 showed the highest average susceptibility (2.90) to scald and F12 the highest resistance (1.36) (Additional file 2: Table S1).

### Marker data HEB-25 parents

According to marker data regarding *Rrs1*, *Rrs2*, *Rrs17* and *Rrs18*, 14 wild barley parents carry at least one positive marker allele associated with known scald resistance genes.

Twelve parents of the HEB families carry resistant marker alleles at *Rrs1*, four carry resistant marker alleles at *Rrs17* and three carry resistant marker alleles at *Rrs18* (Additional file 3: Table S2). Three of them (HID_055 (F03), HID_065 (F05) and HID_144 (F15)) carry resistant marker alleles both at *Rrs1* and *Rrs17*, while HID_138 (F13) carries resistant marker alleles both at *Rrs1* and *Rrs18.* HID_101 (F09) carries resistant marker alleles both at *Rrs17* and *Rrs18.*

The remaining eleven donors including Barke carry no positive alleles of *Rrs1*, *Rrs17* or *Rrs18*. According to the marker results of the diagnostic marker e11_2 no positive *Rrs2* allele is present in the HEB-25 parents.

### GWAS/association

**Fig. 1:**
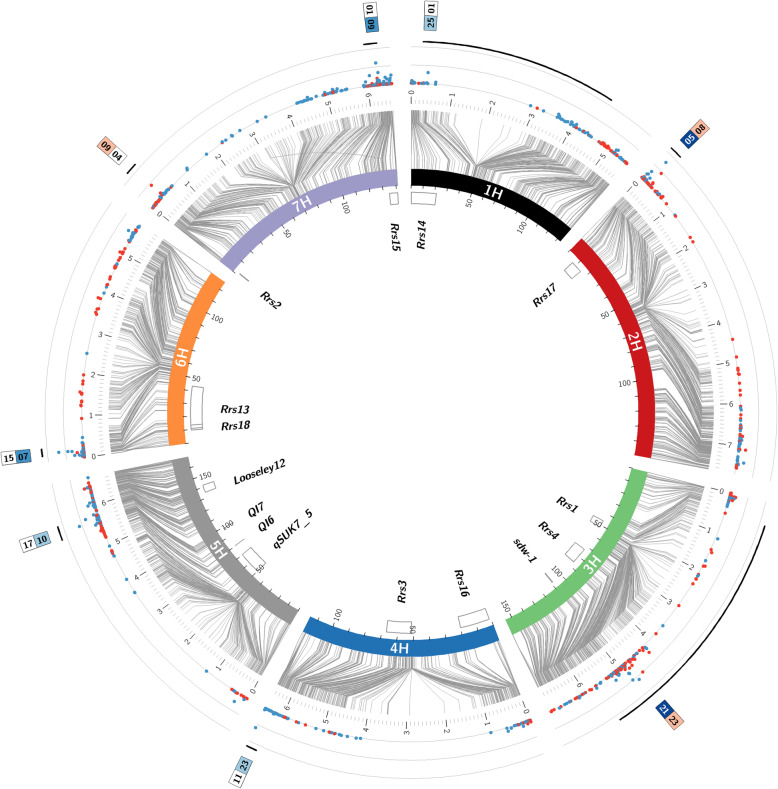
Circos plot indicating QTLs involved in scald resistance. Barley chromosomes are indicated as coloured bars on the inner circle. Grey connector lines represent the link between the genetic position (in cM) of SNPs in the inner circle and the physical position on the outer circle (in Mbp). QTLs and resistance genes from literature are indicated inside the circle and their position is given as outlined boxes on the cM scale. The dots represent the detection rate of each SNP in 100 cross-validation runs with horizontal reference lines at 0, 50 and 100 detections. Red dots represent an average trait-increasing effect, while blue dots represent an average trait-decreasing effect across all HEB families. Black lines on the outer track indicate the range of SNPs on the physical map that have been cumulated for estimating the family-specific effect, which is presented above. Here, the lower box indicates the family with the minimal GWAS effect, while the upper box represents the family with the maximal GWAS effect. The colour code indicates the strength of the effects as a heat map, i.e. darker colour represents a stronger effect. Figure created by use of Circos [58]

**Fig. 2:**
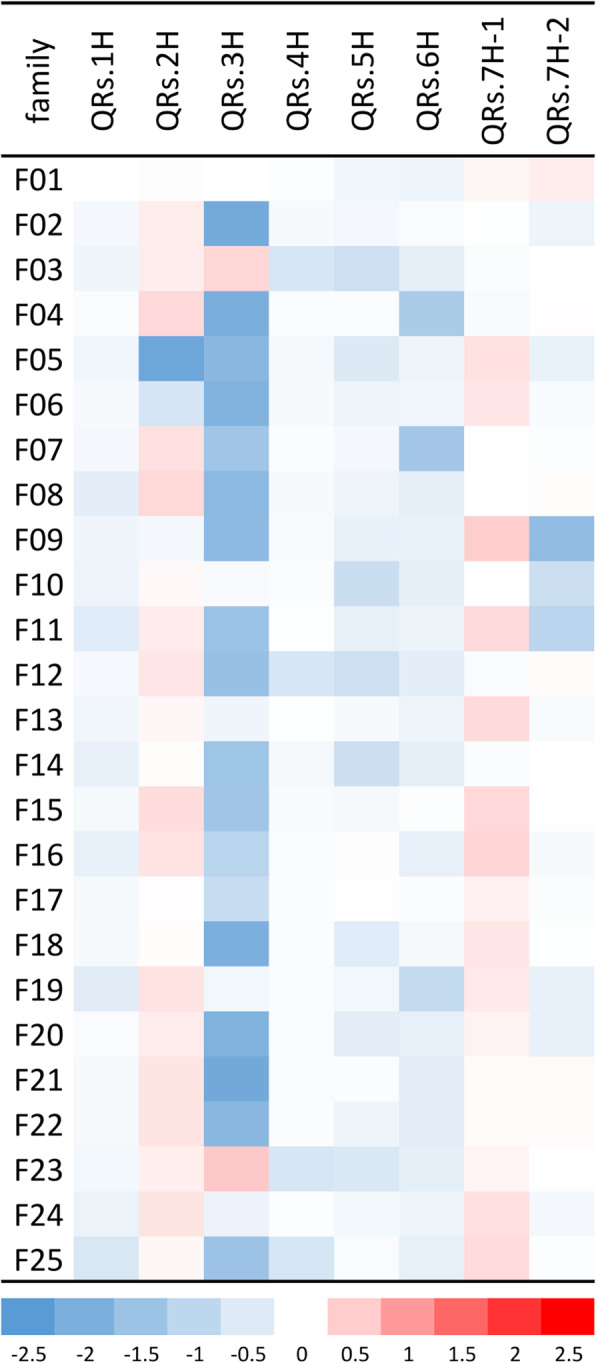
Heat map of family-specific effects at major scald QTLs. For each QTL (columns) GWAS effects of different HEB families (rows) are shown. The colours range from − 2.5 (dark blue) to 2.5 (dark red) scoring units difference as compared to the reference Barke allele. The minimum effect was obtained for F05 at QRs.2H (− 2.22), while the maximum effect was obtained for F23 at QRs.3H (+ 0.54)

**Fig. 3:**
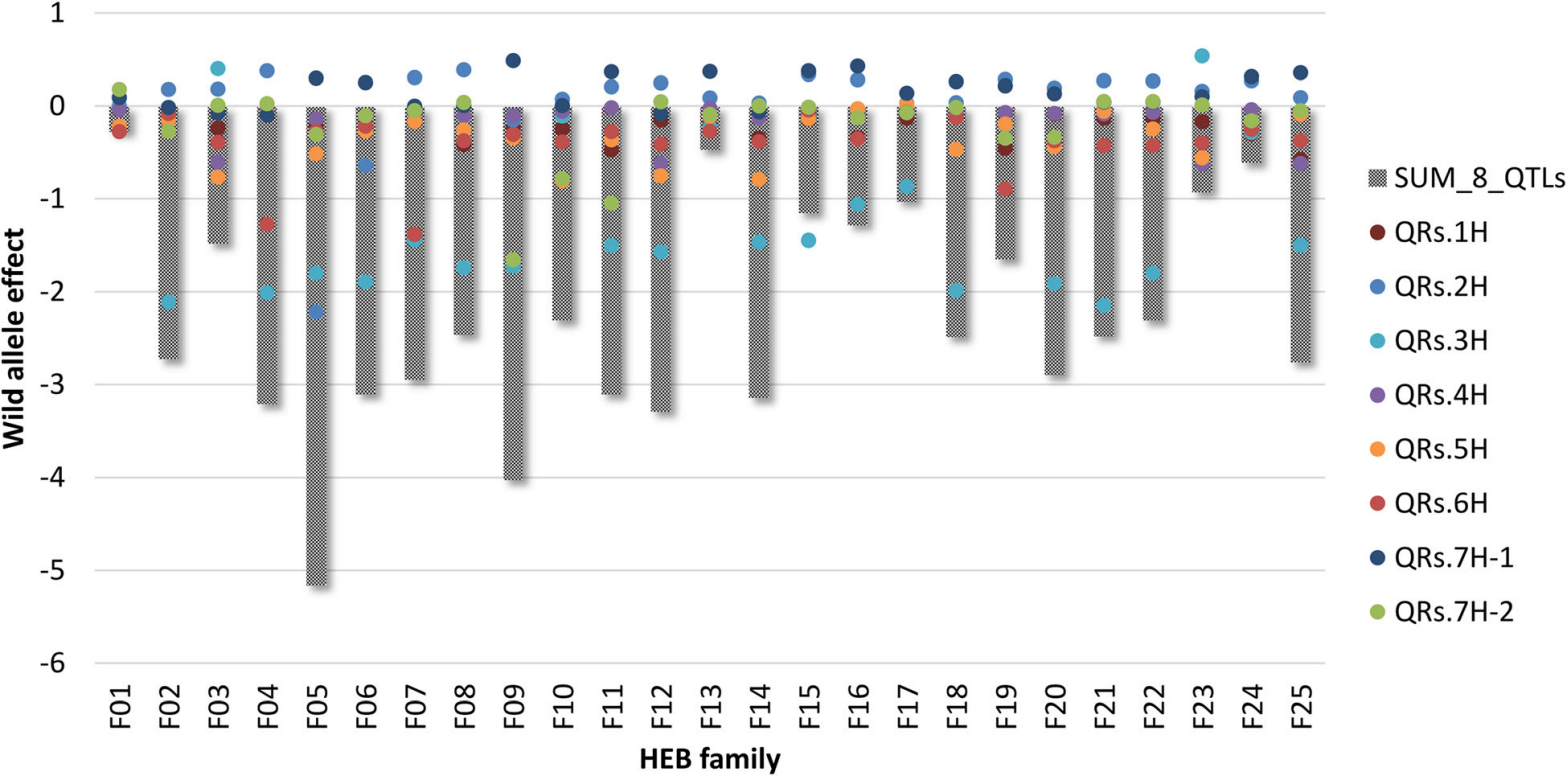
Cumulated donor effect of 8 major scald QTLs. Grey-shaded bars represent the estimated donor effect for each HEB family. Effect was obtained by summarizing family-specific QTL effects of the eight major QTLs, which are represented as coloured dots

NAM revealed eight major QTLs, distributed across all seven barley chromosomes that were reliably detected in more than 30 cross-validation runs (Table 1, Fig. 1). In addition, a few minor QTLs were also detected (Fig. 1, Additional file 4: Table S3). All QTLs together reached a prediction ability (R^2^_val_) of 0.31. QTL effects were highly specific between families. The impact of the wild alleles according to GWAS ranged from a decline in resistance of + 0.54 (QRs.3H (*Rrs1*), F23) to resistance improvement of − 2.22 (QRs.2H (*Rrs17*), F05) scoring units. QTL effects differed strongly at QRs.2H and QRs.3H, indicated by negative and positive wild allele effects revealed in different HEB families. At the most robust QTL (QRs.2H, detected 76 times) the donor of HEB family 5 (F05) had a resistance-improving effect of 2.22 scoring units, whereas almost all remaining donors showed slight resistance-decreasing effects (Fig. 2, Additional file 5: Table S4). The donor alleles at QRs.3H on average showed resistance-improving effects of ≈ 1.7 scoring units in 18 families, whereas F03 and F23 showed comparatively minor resistance-decreasing effects at this QTL. By joint consideration of the eight major QTLs the donor of family 5 showed the most promising resistance effect by improving it by 5.2 scoring units (Fig. 3, Additional file 5: Table S4).

**Table 1:** QTL summary for scald resistance

							# families with GWAS effect^f)^		
QTL	Chr	cM^a)^	bp^b)^	Peak marker^c)^	DR^d)^	R^2 e)^	< 0	< −0.5	CG^g)^
QRs.1H	1H	43.15	38,549,052	BOPA2_12_11266	31	0.08	24	1	
QRs.2H	2H	9.20	15,507,257	JHI_Hv50k_2016_67600	76	0.11	4	2	*Rrs17* [36]
QRs.3H	3H	50.65	481,480,921	JHI_Hv50k_2016_182720	59	0.35	22	18	*Rrs1* [22]
QRs.4H	4H	113.40	646,906,186	JHI_Hv50k_2016_276923	60	0.05	25	4	
QRs.5H	5H	96.98	563,938,261	JHI_Hv50k_2016_321241	58	0.10	24	6	
QRs.6H	6H	28.40	18,911,246	JHI_Hv50k_2016_378176	69	0.09	25	3	*Rrs13* [12]
QRs.7H-1	7H	32.15	38,487,377	JHI_Hv50k_2016_459621	44	0.06	6	0	
QRs.7H-2	7H	111.3	624,196,645	JHI_Hv50k_2016_503391	61	0.12	16	3	

^a^Genetic position in centiMorgan derived from flanking markers based on the barley Infinium iSelect 9 k chip [56]

^b^Physical position based on Bayer et al. [57]

^c^Peak marker of QTL

^d^Detection rate in 100 cross-validation runs

^e^Explained genotypic variance of QTL in the whole NAM population.

^f^Number of families with GWAS effect < 0 (i.e. resistance improving) and < −0.5 (stronger resistance improvement) as compared to Barke

^g^Candidate gene

### Comparison with previously identified genes and QTL

Comparing the identified scald resistance QTLs (Table 1) with QTLs and genes reported in literature (Additional file 6: Table S5) showed that three of the eight identified QTLs are in regions where resistance genes are mapped: QRs.2H co-localizes with *Rrs17*, QRs.3H with *Rrs1* and QRs.6H with *Rrs13*. The two QTLs QRs4.H and QRs.5H are in regions where QTLs have been reported. QRs.1H, QRs.7H-1 and QRs.7H-2 might represent novel QTLs associated with scald resistance as they are distantly located from *Rrs14*, *Rrs2* and *Rrs15*. In the regions of the resistance genes *Rrs4*, *Rrs16* and *Rrs3* no QTL was detected in the HEB-25 population.

## Discussion

To find and describe new alleles of scald resistance the wild barley NAM population HEB-25 was tested for scald resistance against a specific *R. commune* isolate (LfL07A) in controlled greenhouse trials. Eight reliable QTLs were identified, distributed over all chromosomes with two QTLs on chromosome 7H including novel QTLs. The detected variation in resistance in the lines of the HEB-25 infected with *Rhynchosporium commune* reflected a great genetic diversity in the NAM population as shown before for resistance to net blotch (*Pyrenophora teres F. teres*) [52], stripe rust (*Puccinia striiformis f. sp. hordei*) and leaf rust (*Puccinia hordei*) [53].

Most parents of HEB-25 were resistant, only the four parents HID_138 (F13), HID_144 (F15), HID_380 (F24) and Barke (recurrent parent) were susceptible. There were discrepancies between marker genotyping at four known resistance genes described in literature and the phenotyping scores, as for instance HID_138 should carry positive marker alleles at *Rrs1* and *Rrs18* (Additional file 3: Table S2). These discrepancies might be explained, because the markers are not diagnostic (except e11_2 for *Rrs2* [18]), hinting on their limited applicability in wild barley.

Based on the QTL results, for each family the respective donor effect was indirectly derived from accumulating the effects obtained at the eight major QTLs (Fig. 3). According to this the most susceptible donors were observed in F01 (− 0.28 scoring units as compared to Barke), F13 (− 0.47), F24 (− 0.61) and F23 (− 0.93), followed by F17 (− 1.03) and F15 (− 1.15). The direct assessment of scald resistance in the greenhouse revealed the donors of F13, F15 and F24 as the most susceptible ones. The Pearson correlation coefficient of 0.63 between both approaches generally confirms their accordance (Additional file 7: Figure S2). The slight discrepancies for some wild barley accessions (e.g. HID_003 (F01), HID_249 (F17) and HID_359 (F23)) might have been observed due to the heterogenic nature of the wild barley accessions, in the sense that phenotyping was conducted with other plants of the same accession than those being used for initial crossing during HEB-25 development. The segregating susceptibility of HID_144 (F15) between replicates in the greenhouse tests confirms this assumption. The occurrence of heterogeneous seed stocks in genebanks is a common observation and strategies to avoid this are discussed [59].

The most resistant donors according to the cumulated QTL effects are from F05 and F09, followed by F04, F06, F11, F12 and F14. In F05 and F09 all QTLs except QRs.7H-1 have a positive effect on the resistance. Therefore, especially lines from family F05 (e.g. HEB_05_037, HEB_05_041) should be the first choice for breeding to improve scald resistance and to accumulate the respective scald resistance QTLs.

In the following, for each chromosome the detected QTLs are compared to the literature and potential candidate genes are discussed.

### Chromosome 1H

The QTL QRs.1H was detected at a position of 43.15 cM on 1H. All 25 HEB donors except from family F01 have an allele at the QTL that improves resistance, but only in F25 the positive effect is greater than 0.5 rating scores. A known resistance gene on chromosome 1H is *Rrs14,* located in the telomere region of the short arm [19]. In the same region two QTLs linked to the marker Bmac0213 [42, 47] and a meta-QTL [40] were identified. Another QTL (Qsc-1H), distinct from *Rrs14*, was detected by Daba et al. [60]. However, none of the mentioned resistance loci corresponds to QRs.1H from this study. There are 16 high confidence genes with predicted function in the region of the QTL’s peak marker (BOPA2_12_11266) on chromosome 1H (Additional file 8: Table S6). The obvious candidate gene for resistance is the disease resistance protein RGA2 (resistance gene analog 2) [61, 62].

### Chromosome 2H

The QTL QRs.2H in the NAM population is in the region of *Rrs17* (formerly *Rrs15*), which is the only known resistance gene on 2H and is located on the short arm close to the telomere [19, 36]. For QRs.2H (*Rrs17*) only four families showed an improvement in resistance with F05 (− 2.22 scoring units) having the highest impact. This is the highest resistance improvement found in this study. In the region of the QTL on chromosome 2H there are 16 genes (Additional file 8: Table S6), which can play a role in defence, among them three disease resistance proteins [63] and seven kinases [64, 65]. Especially the lectin-domain containing receptor kinase A4.1 [66] and the two LRR receptor-like serine/threonine-protein kinases [67] are clear candidate genes.

### Chromosome 3H

A single QTL (QRs.3H) was found on 3H in the region of *Rrs1* [22]. It represents the main scald resistance QTL in HEB-25 as it explains 35% of genotypic variance. According to GWAS all families except F01, F03 and F23 carry a favourable allele that improves the resistance score up to 2.15 (F21) units compared to Barke. In 18 families the resistance score is improved by more than 0.5 scoring units. Around 50 QTLs are described on 3H, most of them in the region of *Rrs1* (reviewed in [68]). *Rrs1* was linked repeatedly to marker MWG680 [20, 26, 39, 69, 70] and the marker 11_0315 is derived from the same SNP [22]. The 3H QTL identified by Zantinge et al. [71] is linked to seedling and adult plant resistance and located in the region of *Rrs1*_*BC240*_ [70]. The marker identified by Zantinge [71] is located in a gene with a SWAP/Surp (Suppressor-of-White-Apricot) protein domain for which the role in resistance is unknown, other genes identified in the region may play a role in defence, e. g. leucine rich repeats (LLRs) [71]. Recently, a further major QTL for scald resistance at seedling and adult plant growth stage was identified in Yerong [72], confirming a resistance QTL in Yerong identified by Li and Zhou (2011) [42]. It was shown by differential variety screening and physical mapping that the locus is different from *Rrs1* [72]. In our study, from the thirteen candidate genes in the QTL interval on 3H (Additional file 8: Table S6) the two kinases are the obvious candidate genes like on chromosome 2H. Also the beclin-1-like protein [73, 74], the homeodomain-like superfamily protein [75] and the exocyst complex component 7 [76] have been shown to play a role in defence, too.

*Sdw1,* located on the long arm of 3H and causing a semi-dwarf phenotype [77], can influence resistance via escape mechanism, hence resistance QTLs in this region are probably pleiotropic effects of plant height [2]. As expected, no QTL could be detected in this region, because on the one hand only young plants were monitored and on the other hand the advantages of higher plants regarding suppressed spreading of the pathogen is reduced under controlled greenhouse conditions. Also in the region of *Rrs4* [26] on 3H no QTL was detected in HEB-25.

### Chromosome 4H

QRs.4H is not in the region of a known resistance gene like *Rrs3* and *Rrs16* [19], but a QTL has been described in this region before [31, 32, 78]. The powdery mildew resistance gene *mlo* is located in a similar region [32, 34]. The QTL on 4H includes six candidate genes (Additional file 8: Table S6) with a possible role in defence reactions. The disease resistance-responsive (dirigent-like protein) family protein [79] is the obvious candidate. All families have the positive allele slightly improving resistance between 0.02 and 0.62 rating scores compared to Barke, in F03, F12, F23 and F25 greater than 0.5.

### Chromosome 5H

There is no known resistance gene on 5H, but QTLs have been described before corresponding to the Qrs.5H position identified in HEB-25. All HEB-25 families except F18 carry a favourable allele, six of them increase resistance by more than 0.5 scoring units compared to Barke. In the eleven genes in the region of the QTL on chromosome 5H (Additional file 8: Table S6) with a link to resistance again a kinase is found and a cluster of cytochrome P450 superfamily proteins [80].

### Chromosome 6H

The peak marker of QRs.6H falls in the region of *Rrs13* [19] rather than in the closely located *Rrs18* region [38]. All families have the positive allele, the greatest improvement in resistance show F04 and F07 (scores < − 1.0). There are other QTLs in this region [31–35, 69]. As on chromosome 1H and 2H disease resistance proteins [61–63, 72] are found in the region of the QTL (Additional file 8: Table S6) and as on chromosome 2H, 3H and 5H protein kinases [64, 81–83] are plausible candidate genes.

### Chromosome 7H

On 7H the two QTLs QRs.7H-1 and QRs.7H-2 were detected. However, their peak markers do not correspond to *Rrs2* [18] or *Rrs15* [19]. In case of *Rrs2* this is supported by the marker scores of the diagnostic marker e11_2 [18], which indicate the absence of the resistant allele in all HEB families. At QRs.7H-1 most families carry a resistance decreasing allele of up to 0.5 scoring units. In contrast, sixteen families carry a favourable allele at QRs7H.2 with F09 showing the greatest improvement in resistance of 1.65 scoring units compared to Barke, representing a potentially new effective source of scald resistance for future breeding. Of the genes at QRs.7H-2 (Additional file 8: Table S6) the most evident candidate genes are two protein kinases [64, 82]. There are further genes in the region that can play a role in defence like several BTB/POZ domain-containing proteins [84] and two peroxidase superfamily proteins [85, 86]. The QRs.7H-1 region contains ten potential candidate genes with a link to resistance, among them GDSL esterase/lipase [87], ring E3 ubiquitin ligase [88] and acidic endochitinase [89].

## Conclusions

In the NAM population HEB-25 numerous genetic sources of scald resistance could be observed. Both known resistance genes (*Rrs1, Rrs13* and *Rrs17*) and novel QTLs with ample variation among the different wild donors of HEB-25 could be detected. The most impactful resistance allele was obtained at *Rrs17* in HEB family 05. Novel genetic variation that may be utilized in future breeding programs could furthermore be revealed for F09 at a novel QTL on chromosome 7H (QRs.7H-2). However, one has to consider that our results are based on a single *R. commune* isolate. As an outcome of the study the link to the physical map and the identified candidate genes will facilitate cloning the scald resistance genes. Furthermore, the segregating subpopulations of selected HEB-25 BC_1_S_3_ lines can be utilized for fine mapping through the heterogeneous inbred family (HIF) concept [90]. At least the closely linked markers can be used for marker-assisted selection of the underlying resistance genes and marker based introgression of the new identified resistance genes in pre-breeding programs.

## Methods

### NAM population HEB-25

The NAM population HEB-25 [56], consisting of 1420 individual BC_1_S_3_ lines in 25 wild barley derived families, was used in this study. HEB-25 is the result of initial crosses between the elite spring barley cultivar Barke (released in 1996) and 25 highly divergent exotic wild barley accessions (*Hordeum v*. ssp. *spontaneum* and *H. v.* ssp. *agriocrithon*). F_1_ plants of the initial crosses were backcrossed once with Barke. For detailed information about the population design, see Maurer et al. [56]. All seeds were obtained from the Chair of Plant Breeding at Martin Luther University Halle. The exotic wild barley accessions were obtained from the Max Planck Institute for Plant Breeding Research (Cologne, Germany) and their origin is described in Badr et al. [91].

### Resistance assessment in greenhouse

In total, 1379 BC_1_S_3:5_ lines of the NAM population as well as the 26 parents were tested for scald resistance in seven independent greenhouse experiments with four replicates per line. Isolate ‘LfL07A’ of *Rhynchosporium commune* from the collection of the Bavarian State Research Center for Agriculture (Germany) was used to evaluate the resistance level. The fungal isolates were prepared for inoculation as described in Hofmann et al. [22].

The greenhouse test was conducted according to Hofmann et al. [22]. In brief, four plants of each tested line were grown at 18 °C in the greenhouse to the three-leaf stage and then spray-inoculated (in average 280,000 spores/ml). Afterwards plants were kept for 48 h in dark with 100% humidity. Rating on the second leaf started around 14 days after infection and continued three times every other day applying a scale of 0 (resistant) to 4 (susceptible) [19, 92], extended by half steps. The rating score of the third date (18 days post infection) was used as the final phenotypic value, as it represented the time point with the best differentiation between resistant and susceptible lines. Least squares means (LSMeans) were calculated for each HEB line to adjust for the random factor of different experiments with *PROC MIXED* (SAS Institute Inc., Cary, NC, USA) (Additional file 2: Table S1). The reference genotype for phenotyping was the highly susceptible cultivar Beatrix [22], which was highly susceptible in all tests (score 4.0).

### Genotyping

#### Locus-specific markers (HEB-25 parents)

Genomic DNA of the HEB-25 parents was isolated from freeze-dried barley leaves (single plant) according to Behn et al. [93]. To test the linkage between resistance and markers known to be linked to resistance genes the parents of the NAM population were genotyped with the molecular markers 11_1476, 11_0205, 11_0315 [22] and SCRI_RS_221644 [19] for *Rrs1*, e11 for *Rrs2* [18], scsnp07305, STS_2048 and GBS0346 for *Rrs13/Rrs18* [37, 38], and Gems13 for *Rrs15*_CI8288_/*Rrs17* [35, 36] (Additional file 3: Table S2). The marker alleles of the highly susceptible cultivar Beatrix were used as negative references at all marker loci.

#### Genome-wide markers (HEB-25 population)

DNA of pooled BC_1_S_3:8_ plants of each HEB line was extracted according to the manufacturer’s protocol, using the BioSprint 96 DNA Plant Kit and a BioSprint work station (Qiagen, Hilden, Germany), and finally dissolved in distilled water at approximately 50 ng/μl for genotyping with the recently developed barley Infinium iSelect 50 K chip [57] at TraitGenetics, Gatersleben, Germany. SNP markers that did not meet the quality criteria (polymorphic in at least one HEB family, < 10% failure rate, < 12.5% heterozygous calls) were removed from the data set. Altogether, 33,005 SNPs met the quality criteria and were analysed in this study. Fifty-five of the investigated 1379 lines were eliminated due to inconsistent genotypes between BC_1_S_3_ and BC_1_S_3:8_. Based on the Barke reference genotype, the wild barley allele can be specified in each segregating family. To setup the quantitative identity-by-state (IBS) matrix the state of the homozygous Barke allele was coded as 0, while HEB lines that showed a homozygous wild barley genotype were assigned a value of 2. Consequently, heterozygous HEB lines were assigned a value of 1. If a SNP was monomorphic in one HEB family but polymorphic in a second family, lines of the first HEB family were assigned a genotype value of 0, since their state is not different from the Barke allele. Gaps in the genotype matrix resulting from missing data points (0.84%) were filled applying the mean imputation (MNI) approach [94].

The genotype matrix is deposited at e!DAL [95, 96].

### Nested association mapping

We used model ‘IBS-M’ [97], initially introduced as Model-A of Liu et al. [98], a multiple linear regression model with SNP markers being included as main effects using the quantitative IBS genotype matrix scores, to conduct genome-wide association mapping on LSMeans of each HEB line trait performance. The analysis was carried out by means of model selection with SAS PROC HPREG. This procedure can select the best model based on a set of predefined possible factors. In our case, all 33,005 SNPs were initially defined as possible factors. Significant SNPs were then determined by stepwise forward-backward regression. SNPs were allowed to enter or leave the model at each step based on the *p*-value (< 0.001) calculated for the marginal F-test of that term. SNPs included in the final model are hereafter referred to as significant SNPs. A SNP’s effect estimate can be interpreted as the allele substitution effect and represents the regression coefficient of the respective SNP in the final model. Note that all significant SNPs’ effect estimates are modelled at the same time in the final model.

### Cross-validation

A five-fold cross-validation was run 20 times to increase the robustness of the results. For this, 100 subsets were extracted out of the total phenotypic data. Each subset consisted of 80% randomly-chosen HEB lines per family. This set was used as the training set to define significant markers and to estimate their effects, while the remaining 20% of lines were used as the validation set. The phenotypes of the validation set lines were predicted based on marker effects estimated in the training set. Prediction ability (R^2^_val_) was then calculated as the squared Pearson product–moment correlation between the observed and predicted phenotypes of the validation set, while R^2^_train_ represents the model fit of the training set [97].

To define QTL regions, we calculated a SNP marker’s detection rate (DR) as the number of times, out of 100 cross-validation runs, it was included in the final model. Robust QTLs were defined if they were detected more than 30 times.

### Cumulating SNPs to estimate parent-specific QTL effects

To estimate a parent-specific QTL effect from model ‘IBS-M’ we applied the cumulation method as presented in Maurer et al. [97]. This procedure was conducted within each of the 100 cross-validation runs and the mean of them was taken as the final parent-specific QTL effect estimate. Moreover, all SNPs from the respective QTL interval were fitted in a linear model to estimate the QTL’s explained genotypic variance (R^2^) in the whole dataset.

### Comparison with previously identified genes and QTLs, candidate genes, link to physical map

Physical map positions of the barley Infinium iSelect 50 K chip were taken from Bayer et al. [57]. If no position was given the position estimate was derived from markers that revealed the highest linkage disequilibrium to the marker under consideration. In case of 4722 SNPs that were shared between the 9 k [99] and the 50 k chip the genetic positions of the 50 k markers were taken from Maurer et al. [56]. The genetic positions of the remaining markers were estimated based on the genetic positions of physically adjacent markers from the 9 k chip. The position of the major resistance genes against scald were integrated in the map according to Looseley et al. [19]. *Sdw1* was integrated in the map by blasting the *HvGA20ox2* sequence (GenBank: KX611234.1) underlying this locus [100] against the barley genome sequence [101]. Candidate genes for found QTLs were identified via BARLEYMAP [102] (accessed on 25 October 2019), by screening a 2 Mb region surrounding the QTL peak markers for high confidence genes on the physical map [101].

An overview of QTLs conferring resistance including candidate genes described in literature is given in Additional file 8: Table S6.

## Supplementary Information

**Additional file 1: Figure S1**: Overview of scald symptoms.

**Additional file 2: Table S1**: Raw data and LSMeans for scald susceptibility.

**Additional file 3: Table S2**: Genotyping results of HEB-25 parents with locus-specific markers.

**Additional file 4: Table S3**: GWAS results including all markers that were detected in at least one cross-validation run.

**Additional file 5: Table S4**: Family-specific effects of eight major scald QTL defined in GWAS.

**Additional file 6: Table S5**: Summary of reported scald resistance genes and QTLs.

**Additional file 7: Figure S2**: Correlation of real HID phenotype scores and their derived donor effect obtained from GWAS results.

**Additional file 8: Table S6**: Barleymap output of high confidence candidate genes for each of the eight major QTLs.

## Data Availability

All data generated or analysed during this study are included in this published article and its supplementary information files (Additional file 2: Table S1, Additional file 3: Table S2). The genotype matrix is deposited at e!DAL [95, 96].

## Abbreviations

APR: Adult plant resistance
CG: Candidate gene
DR: Detection rate
GWAS: Genome-wide association study
HEB: Halle exotic barley
HID: Hordeum identity (donor of HEB family)
LSMeans: Least squares means
NAM: Nested association mapping
QTL: Quantitative trait locus
