# Supplementary material for: Identification of QTLs conferring resistance to scald (Rhynchosporium commune) in the barley nested association mapping population HEB-25
Source: BMC Genomics. 2020 Nov 27;21:837. doi: 10.1186/s12864-020-07258-7 (PMC7694317; doi:10.1186/s12864-020-07258-7)
Supplement: Supplementary file 1 — Additional file 1: Figure S1: Overview of scald symptoms. [file 12864_2020_7258_MOESM1_ESM.pdf]

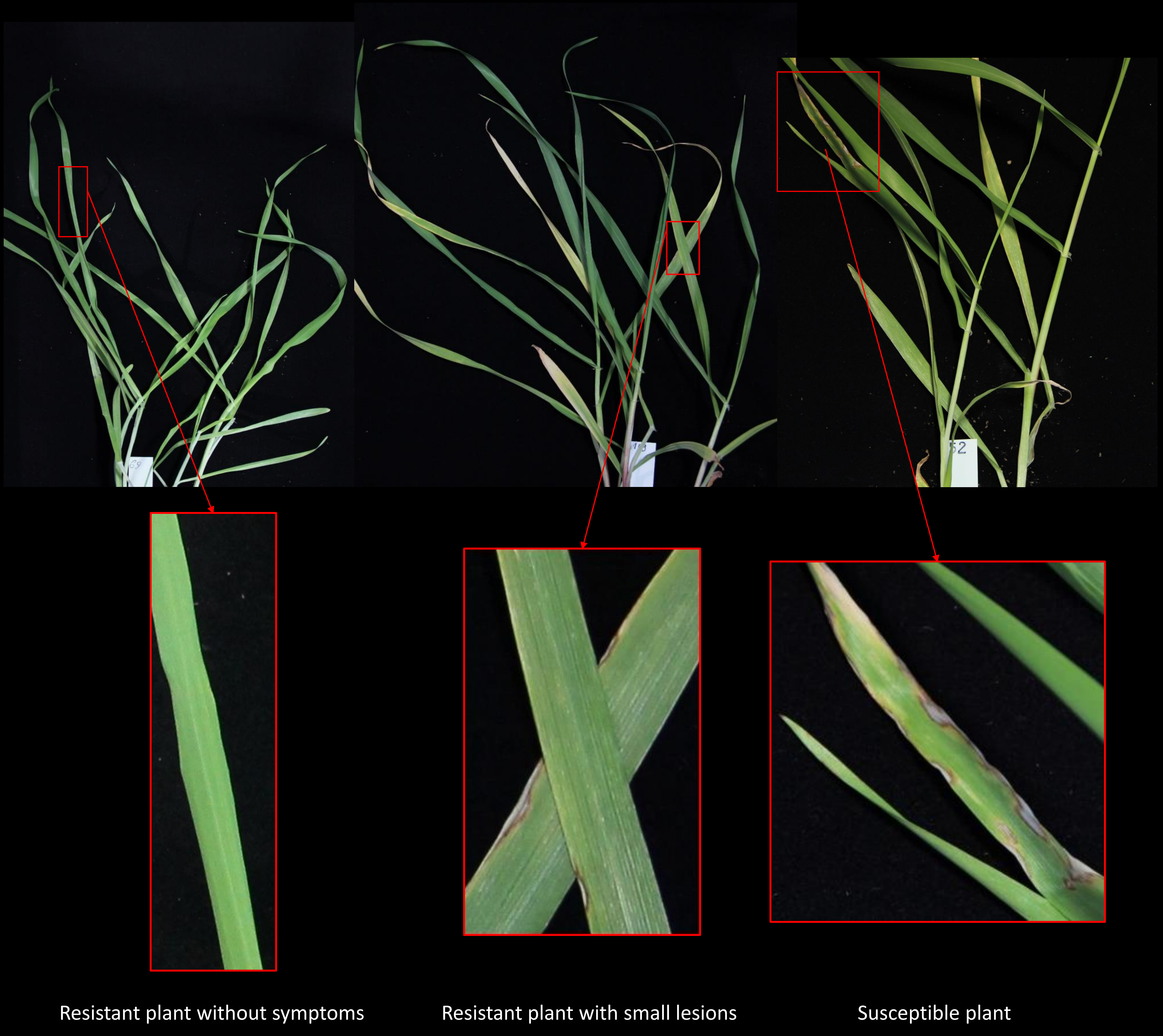

Figure S1. Overview of scald symptoms.
Comparison of phenotypes of a resistant plant without any symptoms (left), a resistant plant with small lesions (middle) and a susceptible plant (right).
