# Supplementary material for: Identification of QTLs conferring resistance to scald (Rhynchosporium commune) in the barley nested association mapping population HEB-25
Source: BMC Genomics. 2020 Nov 27;21:837. doi: 10.1186/s12864-020-07258-7 (PMC7694317; doi:10.1186/s12864-020-07258-7)
Supplement: Supplementary file 7 — Additional file 7: Figure S2: Correlation of real HID phenotype scores and their derived donor effect obtained from GWAS results. [file 12864_2020_7258_MOESM7_ESM.pdf]

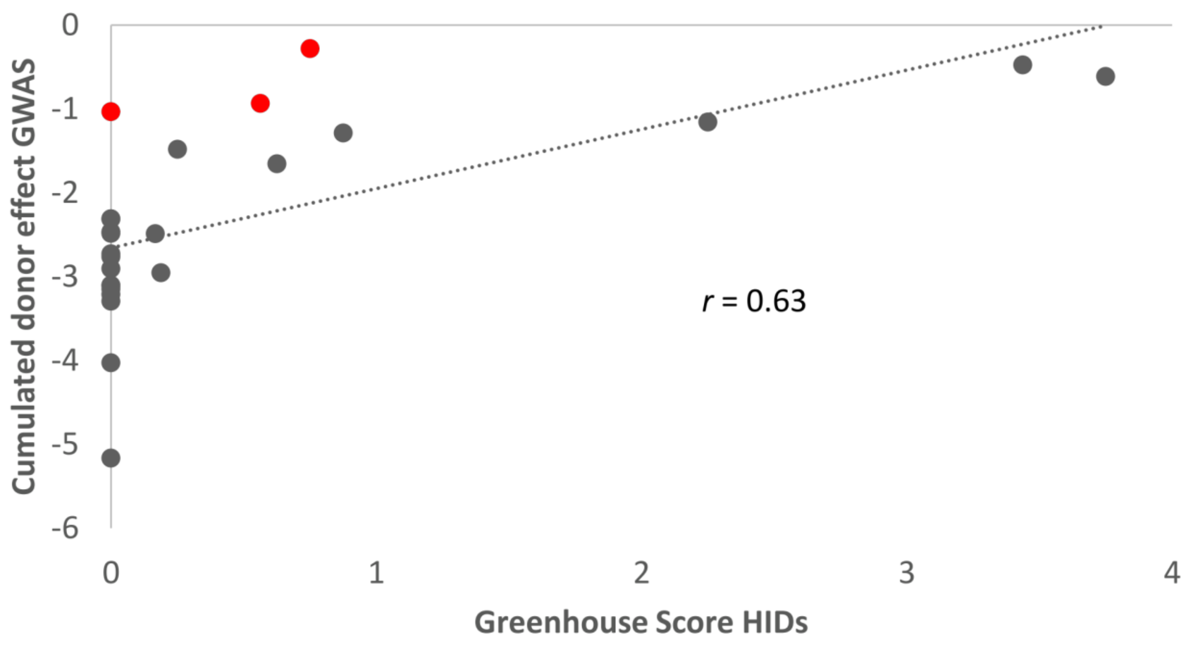

Figure S2: Correlation of real HID phenotype scores and their derived donor effect obtained from GWAS results.
Comparison of average greenhouse scoring for scald resistance (based on Jackson and Webster 1976) and cumulated donor effect as obtained after GWAS for all 25 wild donors. Red dots represent outliers that may be the result of heterogeneous seed stocks that were used for population development and phenotyping. Nevertheless, the overall Pearson correlation between both traits is good (*r* = 0.63).
